# Population-level Impact of Insomnia on Dementia among Older Adults in the U.S.

**DOI:** 10.1093/geroni/igaf122.2227

**Published:** 2025-12-31

**Authors:** Yuqian Lin, Fei Wu, Yuhan Wu, Wanru Liu, Goodarz Danaei, Ruijia Chen

**Affiliations:** Massachusetts General Hospital, Boston, Massachusetts, United States; Harvard T.H. Chan School of Public Health, Boston, Massachusetts, United States; Harvard T.H. Chan School of Public Health, Boston, Massachusetts, United States; Brown University, Providence, Rhode Island, United States; Harvard T.H. Chan School of Public Health, Boston, Massachusetts, United States; Boston University School of Public Health, Boston, Massachusetts, United States

## Abstract

Insomnia has been identified as a plausible modifiable risk factor for dementia, yet no studies have quantified its population-level impact on dementia risk in the United States. Using data from 2022 National Health and Aging Trends Study and relative risks from a published meta-analysis, this study estimated the population attributable fraction (PAF) of dementia cases due to insomnia among U.S. adults aged 65 and older, overall and stratified by age and sex. Among 5,899 participants (44.7% aged ≥80 years; 57.9% female), 28.7% reported having experienced sleep-onset insomnia, sleep-maintenance insomnia, or both. The prevalence of probable dementia was 6.6%, increasing with age for both sexes. The estimated PAF of probable dementia due to any type of insomnia was 12.8% (95% CI: 1.1%, 27.1%), with higher estimates in females (13.4%) than males (12.0%). Among females, insomnia had the largest contribution to dementia in the youngest age group (ages 65–69, 14.4%). For males, the greatest contribution was observed in the 70–74 age group (12.8%). An estimated 471,229 (95% CI: 42,313, 1,014,816) dementia cases in the U.S. in 2022 could have been prevented if insomnia had been eliminated among adults aged 65 and older. Approximately 13% of probable dementia cases, almost half a million cases per year, among older adults in U.S. could be attributable to having any types of insomnia, an estimate comparable to the well-established risk factors such as hearing loss. These findings underscore the potential for interventions aimed at addressing insomnia to reduce dementia risk in aging populations.

